# Treatment options for refractory ulcerative colitis: Small molecules, big effects

**DOI:** 10.1002/ueg2.12585

**Published:** 2024-05-07

**Authors:** Irene Marafini, Joana Roseira, Marjolijn Duijvestein

**Affiliations:** ^1^ Gastroenterology Unit Policlinico Universitario Tor Vergata Rome Italy; ^2^ Gastroenterology Department ‐ unidade local de saúde do Algarve unidade de Portimão Portimão Portugal; ^3^ ABC ‐ Algarve Biomedical Center Faro Portugal; ^4^ Department of Gastroenterology and Hepatology Radboud University Medical Center Nijmegen the Netherlands

Although the majority of patients with ulcerative colitis (UC) have a mild‐to‐moderate disease, approximately 10%–15% experience a severe disease course and require immunosuppressive therapies.^1^ A better understanding of the mechanisms sustaining the pathogenic process in inflammatory bowel diseases (IBD) has largely contributed to expand the therapeutic armamentarium for this group of patients. Alongside with conventional therapies, monoclonal antibodies against tumour necrosis factor‐α, α4β7 integrin (vedolizumab), interleukin (IL)‐12/IL‐23 p40 subunit (ustekinumab), and small molecules inhibiting intracellular pathways downstream to cytokine receptors (tofacitinib, filogotinib and upadacitinib), have entered into the clinic for the treatment of UC.^2^ However, selecting the appropriate medical therapy for each patient at a given stage of the disease natural history is an increasingly complex task for clinicians, as no prediction for treatment effect can be made in the individual patient.

In this context, significant expectations are placed on precision medicine. Personalised approaches to therapy could have the advantages to improve efficacy of a given drug and limit adverse reactions, thereby improving quality of the life of patients and reducing costs. Despite the efforts of the scientific community to identify biomarkers or metabolic/genetic signatures able to predict drug response, personalised therapy in IBD is still in its infancy.^3^ As so, significant focus has been placed on evaluating and comparing the efficacy and safety of existing therapeutic options for UC. Recently, indirect comparisons on therapies' relative efficacy and safety, conducted through network meta‐analysis of randomised control trials (RCTs), were published.^4^ However, RCTs have very strict eligibility criteria which can limit result generalisability to clinical practice, and this was shown to be particularly relevant in IBD.^5^ Therefore, real‐world studies comparing currently available drugs and directing clinicians to the best therapeutic choice are needed.

In this multicentre retrospective study, Allocca and colleagues evaluated the comparative efficacy of tofacitinib and ustekinumab as third‐line treatments for UC patients who have failed both one anti‐TNF and vedolizumab.^6^ The primary objective of the study was to compare the risk of disease progression (defined as the need of steroid and/or therapy escalation, hospitalisation and UC‐related surgery) over time between the ustekinumab and tofacitinib treatment groups. Clinical remission, normalisation of C‐reactive protein, endoscopic remission and treatment withdrawal were secondary outcomes. Sixteen centres across Europe and Israel were involved in the study, 117 patients were enrolled and followed‐up for a median time of 11.6 months, 41 patients (35%) received ustekinumab and 76 patients (65%) received tofacitinib.

With regard to the primary outcome, it is noteworthy that treatment with ustekinumab was associated with a significant increased risk of disease progression compared to tofacitinib: 28 patients (68%) in the ustekinumab group and 35 (46%) in tofacitinib group had disease progression. In the multivariate logistic regression analysis no differences were observed between the two treatment groups regarding the secondary outcomes. The rate of adverse events was numerically higher in the tofacitinib group compared to ustekinumab.

Overall, the data from this study suggest that tofacitinib can be considered before ustekinumab in the treatment algorithm of patients with refractory UC in the absence of factors that contraindicate its use.

Despite the retrospective nature of the study, the methodology employed was rigorous particularly given the complexities associated with retrospective observational studies in a multicenter context. Also, the adoption of time‐to‐event analysis and the use of Kaplan–Meier survival curves, complemented by Cox proportional hazards modelling, are adequate choices for evaluating longitudinal treatment effects in a clinical population that exhibits wide variability in response to treatment. With regard to safety, it is worth emphasising that the short follow‐up does not allow to draw definitive conclusions given the possibility of missing serious adverse events that may occur in the long term.

A recent retrospective cohort study in a real‐world setting showed that tofacitinib and ustekinumab had similar effectiveness in inducing steroid‐free clinical remission at 52 weeks, along with comparable drug persistence in UC patients after anti‐TNF failure.^7^ These findings are consistent with those of Allocca et al., who also reported equivalent clinical efficacy between the two treatment groups. However, symptom‐based scoring assessments have gradually been losing ground in UC. Importantly, Allocca and colleagues incorporated a composite outcome that reflects disease progression, a crucial aspect given the recognition of UC as a progressive disease. This comprehensive approach allowed to demonstrate the superiority of tofacitinib regarding this specific outcome, marking a significant step forward in understanding its therapeutic impacts in UC.

This multicenter real‐world study has the great merit to attempt to fill a significant gap in the literature concerning drug sequencing and efficacy in a highly specific subgroup of patients (failing both one anti‐TNF and vedolizumab).

Although further large‐scale prospective real‐world studies and randomised‐controlled trials are necessary to validate these findings, this study serves as a prime example of what the IBD community requires: multicenter, well‐designed research, addressing precise clinical questions that have the potential to enhance clinical practice.

## CONFLICT OF INTEREST STATEMENT

IM received speaking fees from Galapagos, Abbvie; JR received speaking fee from Abbvie and Janssen; MD received Speaking fee from Bristol Meyers Squibb, Takeda, Galapagos, served in an advisory board for Abbvie, Bristol Meyers Squibb, Celltrion, Galapagos, Janssen, Takeda, received Grant/Research support from Pfizer, Bristol Meyers Squibb, Galapagos and Janssen.
